# Deep-Compact-Clustering Based Anomaly Detection Applied to Electromechanical Industrial Systems

**DOI:** 10.3390/s21175830

**Published:** 2021-08-30

**Authors:** Francisco Arellano-Espitia, Miguel Delgado-Prieto, Artvin-Darien Gonzalez-Abreu, Juan Jose Saucedo-Dorantes, Roque Alfredo Osornio-Rios

**Affiliations:** 1 MCIA Department of Electronic Engineering, Technical University of Catalonia (UPC), 08034 Barcelona, Spain; miguel.delgado@upc.edu; 2 HSPdigital CA-Mecatronica Engineering Faculty, Autonomous University of Queretaro, San Juan del Rio 76806, Mexico; agonzalez63@alumnos.uaq.mx (A.-D.G.-A.); jsaucedo@hspdigital.org (J.J.S.-D.); raosornio@hspdigital.org (R.A.O.-R.)

**Keywords:** condition monitoring, anomaly detection, deep neural networks, autoencoder, compact clustering

## Abstract

The rapid growth in the industrial sector has required the development of more productive and reliable machinery, and therefore, leads to complex systems. In this regard, the automatic detection of unknown events in machinery represents a greater challenge, since uncharacterized catastrophic faults can occur. However, the existing methods for anomaly detection present limitations when dealing with highly complex industrial systems. For that purpose, a novel fault diagnosis methodology is developed to face the anomaly detection. An unsupervised anomaly detection framework named deep-autoencoder-compact-clustering one-class support-vector machine (DAECC-OC-SVM) is presented, which aims to incorporate the advantages of automatically learnt representation by deep neural network to improved anomaly detection performance. The method combines the training of a deep-autoencoder with clustering compact model and a one-class support-vector-machine function-based outlier detection method. The addressed methodology is applied on a public rolling bearing faults experimental test bench and on multi-fault experimental test bench. The results show that the proposed methodology it is able to accurately to detect unknown defects, outperforming other state-of-the-art methods.

## 1. Introduction

The new era of a smart manufacturing environment is characterized by the rapid development of industrial technology, information systems and components of industrial systems becoming increasingly complex. Consequently, industrial systems are required to be safe and reliable in order to attend production demands. In this sense, the implementation of information technology in production processes is increasingly on the rise. Considering that extensive information collected from multiple monitoring sensors has been generated and the increasing capacity to process data using artificial intelligence algorithms have brought great potential for the implementation and build-up of data-driven condition monitoring (DDCM) approaches [1]. However, some of the challenges that DDCMs facing to smart manufacturing environment include: (1) having a high capacity for pattern management, (2) be adaptive to the complexity of the systems, (3) as well as to the different operating conditions and the occurrence of faults on different components. In this regard, multiple DDCM approaches have been proposed for fault diagnosis in industrial systems. For example, Manjurul, et al. [2], proposed a scheme of feature models for fault diagnosis of bearings using multiclass support vector machines (SVMs). Likewise, in [3], a multi-faults diagnosis method based on high-dimensional feature reduction and artificial neural networks is utilized. Furthermore, Rauber, et al. [4], proposed a methodology based on feature extraction and dimensionality reduction with principal component analysis applied to bearing fault diagnosis. Another relevant studies that address the basic guidelines of DDCM approaches on machine learning are [5,6]. Although this intelligent fault diagnostics research reports a significant advance in predictive maintenance, there are still some limitations. On the one hand, the performance of classical methods based on machine learning is poor when it comes to complex systems due to their limited ability to characterize multiple patterns [7,8]. On the other hand, these approaches do not consider the behavior of previously unseen patterns. These uncharacterized patterns may be deviations from system due to the evolution in the useful life of the machinery under supervision, the presence of new fault scenarios and/or the capability of increasing the knowledge to assess additional severities of faults that have been already identified.

To address the challenge of pattern management, deep learning (DL) techniques have recently been widely applied for fault diagnosis methods over electromechanical systems [9,10]. Note that the term pattern management is considered a good characterization and extraction of features in this work. By the implementation of DL, it is possible to extract the complex relationships from the data through the use of neural networks with multiple layers and non-linear transformations [11]. The multilayer approach represents an advantage since it allows to deepen the characterization of patterns in an effective and adaptive way, unlike the handcraft feature extraction process of ML-based algorithms [12]. Some examples of the use of DL in the field of condition monitoring in modern electromechanical systems are based on deep neural networks (DNN) [13]; based on convolutional neural networks (CNN) [14]; based on long short-term memory (LSTM) [15]; and based on deep-autoencoders (DAE) [16]. However, as a trade-off between performance and its adaptability to time data, usually used for condition monitoring, the use of DAE has been widely used [17,18,19,20,21,22].

Despite the fact that these DL-based approaches are extremely successful in pattern management and diagnosis, that is, fault classification, there is an important issue that has not been clearly solved in regard to how to consider the detection of unseen patterns. Therefore, the detection of unseen patterns is a topic that attracts the attention of researchers in the field of pattern recognition, and especially in the field of industrial systems prognosis. It is a real problem, that when mistakes are made during the condition assessment, it has a negative impact, especially to early decision-making and an incorrect diagnosis that can lead to considerable losses or catastrophic faults. Recently, some approaches based on domain adaptation have addressed the issue of characterizing patterns that are unseen or that present some type of imbalance between the operating conditions of the training and testing data [23,24,25,26]. Despite this, several approaches have been successfully proposed to deal with the problems of unseen patterns in machinery; most of these proposals have been designed under semi-supervised schemes, and not all of them are adapted to operate online.

Due to in a real industrial environment only the normal (healthy) class is available as initial knowledge, it is necessary to adapt approaches that consider the use of one-class classifiers. One-class classification (OCC) problems are sometimes referred to as anomaly detectors, novelty, or outliers [27,28,29]. These are trained with known patterns that are organized as one or more groups (in feature space) of normal classes. The model is then used to identify unknown patterns such as novelties or anomalies [30], which are somewhat different from those present in the original training data set.

There are different schemes to carry out OCC problems. One of them is based on estimating the generative probability density function (PDF) of the data, such as the Gaussian mixture model (GMM) [31]. Distance-based methods, include for example, nearest neighbors [32]. Another approach is based on a reconstruction model. The autoencoders (AE) are examples of this type of approach [33]. Domain-based methods impose a boundary to be created based on the structure of the training dataset. In this case, a limit optimization problem is solved to represent the data. Class membership of anomalous data is then determined by their location with respect to the boundary. One-class support vector machine (OC-SVM) and support vector data description (SVDD) are the most popular methods [34]. In this regard, OC-SVM is the approach that has been used the most and that improves performance as a trade-off between simplicity of configuration and performance shown when adapting to the representation of the data [35].

**Figure 1:**
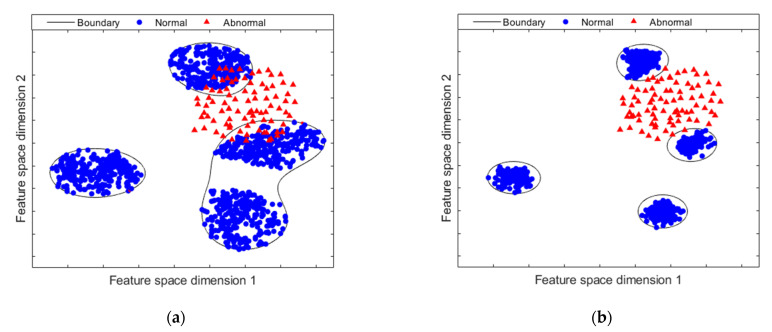
Feature space mapping: (**a**) The feature space mapping without a compact representation shows a high overlap between both classes; (**b**) feature space mapping with an improved and compact representation. It shows a higher separation between normal and abnormal samples.

However, for real-world problems, regardless of their approach, there are other problem to be addressed: the distribution and behavior of the characterized patterns. It should be noted that, in industrial operation environments, the behavior of the patterns tends to have a sparse representation, that is, the distribution of the known class can be represented by more than one cluster within the features space. Taking into account that for the OCC-based approach to have a better result, the normal class distribution must be as compact as possible. The assumption that a compact feature space improves the performance of OCC classifiers is shown in Figure 1. In Figure 1a shows a feature space without a compact representation. In this case, the samples of the normal and abnormal class can overlap. As a consequence, the classification performance decreases in the presence of a large number of false negatives. Meanwhile, a more compact feature space is shown in Figure 1b. The clusters of the different distributions of the normal class are grouped together. Further-more, there is a wide margin of separation between normal samples and anomalous samples and therefore outside the classification boundary. This effect on performance improvement is also addressed in [36].

Recently, deep-learning methods, such as the convolutional neural networks (CNNs) and deep-autoencoders (DAEs) that have better representation capabilities have been widely applied in many approaches to improve clustering tasks, that is, to achieve better representations of the data [7,37]. Deep-learning-based clustering approaches have been called deep-clustering. Peng et al. [38] proposes a model of feature transformation using DNN. In [39], the authors present a model for clustering based on DL. Xie et al. [40] propose deep embedded clustering (DEC). A clustering algorithm that aims at finding learn representations and cluster assignments using deep neural networks, by reinforcing the compactness and increasing clusters separability. The clustering algorithm presented in [40] was adapted to the OCC context in [41], resulting in relevant improvements for experiments in the field of image detection.

Inspired by the high capacity to pattern management through DL, in clustering-based algorithms and the anomaly detection abilities of OCC schemes, in this paper a novel method called the deep-autoencoder-compact-clustering one-class support-vector machine (DAECC-OC-SVM) is proposed for anomaly detection on electromechanical industrial systems. The main objective of this paper is to propose a methodological process to deal with the problems of DDCMs in industrial systems that are present in the current smart manufacturing environment. The contributions of this paper are summarized below:

1. The proposal of a novel methodological process to carry out the detection of anomalies applied to industrial electromechanical systems.
2. The proposal consists of a hybrid scheme that combines the ability to characterize and extract features from DL with the ability to identify anomalies from an OCC-scheme based on ML. In addition, this scheme is adaptive since it can be applied to any environment of electromechanical systems.
3. The deep embedding clustering proposed in [40] is extended and adapted to the OCC context. A compact representation for anomaly detection applied to electromechanical systems is learned and such representation ends up significantly increasing the final classification performance.

This paper is organized as follows. Section 2 presents the theoretical background focused on deep-autoencoder with compact clustering. Section 3 describes in detail the proposed methodology. Section 4 addresses the validation and analysis. Finally, Section 5 reports the general conclusions.

## 2. Theoretical Background

Clustering is a relevant topic in machine learning and data mining. With the fast growth of the deep neural networks for its exceptional capability to learning non-linear representations [7], recent researches have focused on learning good representations for clustering tasks [42]. This approach is mainly inspired by deep-autoencoder and deep clustering for representation learning. In this part, we focus on these two aspects.

### 2.1. Deep-Autoencoder

The autoencoder is a type of unsupervised fully connected one-hidden-layer neural network that learns from unlabeled datasets. The goal is that the AE is trained to reconstruct the input patterns at the output of the network. An AE takes an input x and transforms it to the latent representation h. This is done using a non-linear mapping function $h=f\left(W_{e}x+b_{e}\right)$, where and f is the non-linear activation function, $W_{e}$ and $b_{e}$ are the weights matrix and biases vector, respectively. For reconstructing the input, a reverse transform $y=f\left(W_{d}h+b_{d}\right)$ is used. The parameters $W_{e}$ and $b_{e}$ learned from the input layer to the hidden layer define the encoder process, and the parameters $W_{d}$ and $b_{d}$ learned from the hidden layer to the output layer define the decoder process.

The training process of the AE consists of the optimization of parameters $\theta_{AE}=\{W_{e},\;b_{e},\;W_{d},\;b_{d}$} to reduce the reconstruction error between the input and the output by measuring the following cost function:

(1) $$E\left(x,y\right)=\Omega_{Mse}+\lambda *\Omega_{weights}+\beta *\Omega_{sparsity}$$

where the first term is the reconstruction error computed as $\Omega_{Mse}=\frac{1}{N}\overset{N}{\underset{k=1}{\sum }}||x_{k}-y_{k}{||}^{2}$, for N data samples, the second term is the L2 regularization term that prevent overfitted responses on AE networks, is defined as follows:

(2) $$\Omega_{weights}=\frac{1}{2}\sum_{i=1}^{a}\sum_{j=1}^{b}\sum_{k=1}^{c}{\left(W_{jk}^{i}\right)}^{2},\;$$

where a is the number of weight parameters, b is the number of rows and  c is the number of columns in each weight matrix W, and $W_{jk}^{i}$ represents each element of W.

$\Omega_{sparsity}$ is the sparsity regularization, and λ is the term for the L2 regularization that controls the weight decay and β is the term for the sparsity regularization term. The sparsity regularizer term is introduced to generate network models with more specialized learning.

This regularizer term is a function of the average output activation value of a neuron. The average output activation measure of a neuron i is as follows:

(3) $$\hat{\rho_{i}}=\frac{1}{n}\sum_{j=1}^{n}f\left(w_{i}^{T}x_{j}+b_{i.}\right),\;$$

where n is the training samples. $x_{j}$ is the jth training sample, $w_{i}^{T}$ is the ith row of the weight matrix $W_{ie}$, and $b_{i.}$ is the  ith input of the bias vector, $b_{e}$. Therefore, a sparsity function term has as purpose to restrict the magnitude of $\hat{\rho_{i}}$ to be sparse, bring on the AE to learn a mapping, so each neuron in the hidden layer be activated to a short number of samples. With the aim that neurons become sparse, we can add a term to the cost function that constrains the magnitude of $\hat{\rho_{i}}.$ The sparsity regularization term is given by the Kullback-Leibler divergence [43], which is used to measure the distance from a desired distribution, where ρ is the expected sparsity parameter and $\hat{\rho_{i}}$ is the effective sparsity of a ith hidden neuron. This function term is as follows:

(4) $$\Omega_{sparsity}=\sum_{i=1}^{n}KL(\rho ||\hat{\rho_{i}})=\sum_{i=1}^{n}\rho \log\left(\frac{\rho }{\hat{\rho_{i}}}\right)+\left(1-\rho \right)\log\left(\frac{1-\rho }{\hat{1-\rho_{i}}}\right)$$

**Figure 2:**
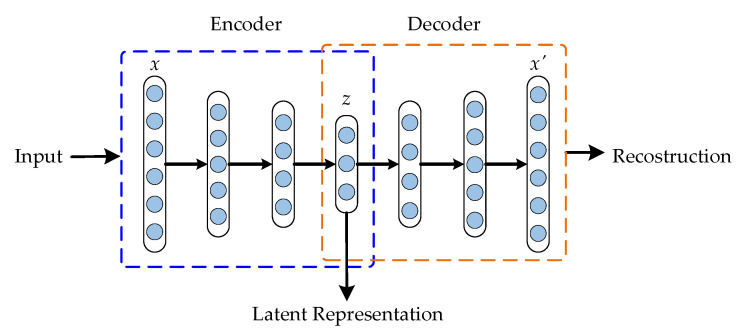
Schematic Deep-Autoencoder Construction.

After training the first AE, its encoder output h is used as input to train the next AE. Afterwards we perform layer-wise training, which concatenates all encoder layers followed by all decoder layers, in reverse layer-wise training order, to build a DAE, as shown in Figure 2. The final result is a multilayer DAE with a bottleneck coding layer in the middle (latent representation or feature space).

### 2.2. Deep-Compact-Clustering

The deep-compact-clustering (DCC) introduced in this work follows the idea presented in [40]. In this, its propound the issue of clustering a set of n points ${\left\{x_{i}\in X\right\}}_{i=1}^{n}$ of a feature space into k clusters, each represented by a centroid $\mu_{j},\;j=1,\ldots ,k.$ Instead of clustering directly in the data space X, its propose to first transform the data with a nonlinear mapping $f_{\theta }:X\to Z$, where θ are learnable parameters and Z is the latent feature space. Therefore, the parameters of $f_{\theta }$ are constructed using data mapped by the bottleneck coding layer of a DAE, discarding the decoder layers.

The DCC approach simultaneously learns a set of k cluster centers ${\left\{\mu_{i}\in Z\right\}}_{j=1}^{k}$ in the feature space Z and the parameters $\theta =\{W_{e},\;b_{e}$} of the encoder layer of DAE.

From the mapping generated by the DAE’s encoder and given the initial k cluster centers, the main idea is to interchange, iteratively, between two principal steps: (1) measure a soft assignment between the embedded points and the cluster centroids; (2) compute an auxiliary target distribution, based on learning the current high-confidence assignments, then, update the deep mapping θ and improve the cluster centroids. To accomplish this, the optimization process is carried out minimizing the Kullback-Leibler divergence loss between soft assignments $q_{ij}$ and the auxiliary target distribution $p_{ij}$:

(5) $$L=KL(P|Q)=\sum_{i}\sum_{j}p_{ij}log\frac{p_{ij}}{q_{ij}}$$

The soft assignment is defined as the probability of assigning sample i to cluster j, using the Student’s t-distribution as a kernel to measure such similarity, following [44].

(6) $$q_{ij}=\frac{{\left(1+{\left|\left|z_{i}-\mu_{j}\right|\right|}^{2}/\alpha \right)}^{-\frac{\alpha +1}{\alpha }}}{\sum_{j^{\prime }}{\left(1+{\left|\left|z_{i}-\mu_{j^{\prime }}\right|\right|}^{2}/\alpha \right)}^{-\frac{\alpha +1}{\alpha }}}$$

where $z_{i}=f_{\theta }\left(x_{i}\right)\;\in \;Z$ correspond to $x_{i}\;\in \;X\;$ after embedding, α are the degrees of freedom of Student’s t-distribution. The auxiliary target distribution is calculated using soft assignments with the following relationship:

(7) $$p_{ij}=\frac{q_{ij}^{2}/f_{j}}{\sum_{j^{\prime }}q_{ij^{\prime }}^{2}/f_{j^{\prime }}}$$

where $f_{j}=\underset{j^{\prime }}{\sum }q_{ij}$ are soft cluster frequencies. The choice of target distributions *p* is the most relevant step in achieving compactness for each group centroid. According to [40], this distribution must satisfy the following concepts: generate good predictions (improve cluster purity), give greater importance on data points assigned with high confidence, and normalize the loss contribution of each centroid to avoid large clusters distort the embedded feature space.

The cluster centers $\mu_{j}$ and DAE parameters θ are then optimized jointly using the stochastic gradient descent method with momentum, and the standard backpropagation to compute parameters’ gradients, which are defined as:

(8) $$\frac{\partial L}{\partial z_{i}}=\frac{\alpha +1}{\alpha }\sum_{j}{\left(1+\frac{{\left|\left|z_{i}-\mu_{j}\right|\right|}^{2}}{\alpha }\right)}^{-1}\times \left(p_{ij}-q_{ij}\right)\left(z_{i}-\mu_{j}\right)$$

(9) $$\frac{\partial L}{\partial \mu_{j}}=-\frac{\alpha +1}{\alpha }\sum_{i}{\left(1+\frac{{\left|\left|z_{i}-\mu_{j}\right|\right|}^{2}}{\alpha }\right)}^{-1}\times \left(p_{ij}-q_{ij}\right)\left(z_{i}-\mu_{j}\right)$$

The optimization continues iteratively until the stop criterion is met.

## 3. Methodology

**Figure 3:**
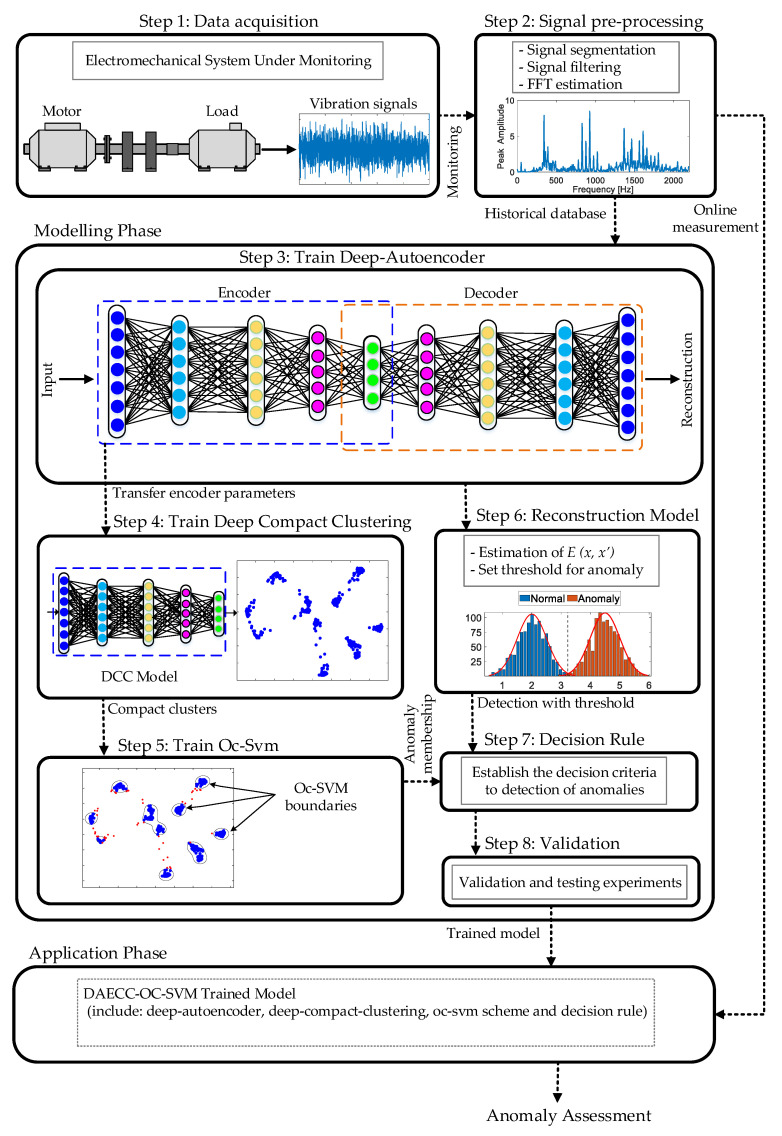
Framework of the proposed method. Step-by-step detailed flowchart of the proposed anomaly detection monitoring methodology.

The framework of the proposed DAECC-OC-SVM method is illustrated in Figure 3. The proposed anomaly detection method can be divided into the following two phases: (i) modelling phase (the offline procedure) and, (ii) application phase (the online procedure). The modelling phase uses available monitoring data (i.e., historic, ad-hoc acquisitions, etc.), to face a data-driven training in which the optimization of the models’ parameters that compose the developed methodology is carried out in an off-line mode. Once the DAECC-OC-SVM method has been trained and validated, it is then ready to be integrated in the on-line operation over the electromechanical system under supervision. Thus, continuously, for each collected sample (i.e., an acquisition of the considered physical magnitudes), the DAECC-OC-SVM method outputs an assessment of whether the new measurement corresponds to a known condition. This provides criteria for making a maintenance decision. In other words, if the new sample is known, the complementary diagnostic systems (outside the scope of this study) can be reliably executed. If it is unknown, the system under monitoring is operating in different conditions than those characterized and, therefore, no more information can be contributed to the maintenance decision-making process than the operating anomaly itself. The procedures of applying DAECC-OC-SVM are divided into two, those corresponding to the offline procedure and the online procedure. Data acquisition and signal pre-processing agree to both phases. Train deep-autoencoder, train deep-compact-clustering, train oc-svm scheme, reconstruction model, decision rule and validation are part of the offline process. Once these steps are completed, the online process is ready to execute and to evaluate the new measurements of the system under monitoring. All these steps are detailed below.

### 3.1. Step 1: Data Acquisition

The first stage of the proposed methodology is related to the collection of information associated to the condition of the rotating system. For this aim, three considerations need to be addressed. First, the acquisition of physical magnitudes, second, the details of the acquisitions and finally, the conditions of the electromechanical system. For the acquisition of physical magnitudes, the proposed method focuses on the acquisition of vibrations from multiple points of the electromechanical system. However, the proposed methodology would allow adaptation to other sources of information, such as stator current, acoustic emission, speed, among others. The proposed methodology is based on the characterization of physical magnitudes for one-second segments. In this regard, considering static behaviors in this period of time, this acquisition time ensures sufficient statistical consistency in most practical applications (i.e., rotation speed greater than 500 rpm). However, the acquisition time can be increased in order avoid the losing of performance for those low-speed applications. For the conditions of the electromechanical system, the present methodology is designed to start with data corresponding to the healthy operating condition, including torque and speed variants that may be presented. In any case, as long as the acquisition details are the same, acquisitions corresponding to different operating states (i.e., faults and degradation levels) can be added. Therefore, the result of this step is a raw vibration signal.

### 3.2. Step 2: Signal Pre-Processing

The vibration signal is then segmented by time-windows with equal length with the aim to generate a set of consecutive samples. The process of decomposing the signal into segments is expressed as follows:

(10) $$X_{vib}=\left[X_{vib}^{1:L},\;X_{vib}^{L+1:2L},\ldots ,\;X_{vib}^{\left(n/h-1\right)h+1:n}\right]$$

where *L* denotes to the length of the time window used for segmentation and *n* denotes the sampling number. With the segmentation process, the vibration signal is divided into *n*/*L* segments.

On the other side, under an industrial environment, the generation of significant noise levels appear due to the inherent operation of rotating systems; as a consequence, the nature of the acquired signal is affected, and the use filtering stages is necessary. Thereby, this proposal considers the implementation of a pre-processing stage that aims to filter a specific range of frequencies of interest, in which, the electromechanical system under study, may generate the fault-related frequency components. Specifically, the considered filter is a digital FIR-based low pass filter that is implemented in software, and it is designed under the window method [45]. The digital low pass filter based on the windowing method works the under basic principle of the ideal “brick wall” filter with a cut-off frequency of $\omega_{0}\;\left(\mathrm{rad}/\sec\right)$ following:

(11) $$h\left(n\right)=\frac{1}{2\pi }\int_{-\omega_{0}}^{\omega_{0}}e^{j\omega n}d\omega$$

where, the low pass filter produces a magnitude equal to one for all frequencies that have frequency values less than $\omega_{0}$, and produces a magnitude equal to 0 for those frequency values between $\omega_{0}$ and π. Thus, its impulse response sequence is depicted by h(n).

Therefore, the main objective of this filtering process is to obtain the vibration signal in a suitable form for further processing; in this sense, each segmented part of the signals $X_{vib}$ have been individually subjected to the low pass filtering stage with a cut-off frequency equal to 1500 Hz. After the filtering process, spectra of the signals are obtained using fast Fourier transform (FFT). Therefore, the corresponding frequency amplitude is obtained for each signal segment. Finally, the frequency coefficients are scaled and used as the final inputs of the model. Since the frequency amplitude is too small to cause the change of network weights, the samples are multiplied by a coefficient following the recommendations of [46]. Regarding the dataset, it has been divided in three different parts: the first one composed of 70% of the available samples for training purposes, the second one composed of 20% of the samples for validation purposes, finally, the third one composed of 10% of the samples for test purposes. In addition, a five-fold cross-validation approach is applied over the data in order to corroborate that the results are statistically significant. In this step, the result is segments of vibration signals filtered and transformed into frequency components.

### 3.3. Step 3: Train Deep-Autoencoder

The DAE architecture proposed by [47], trained layer by layer is adopted in this pa-per. The DAE hyperparameters, such as, the coefficient for the L2 regularization term, the coefficient for the sparsity regularization term and, the parameter for sparsity proportion, as well as the number of neurons in each hidden layer, are established from the search for the optimal configuration using a genetic algorithm (GA).

The optimization procedure is performed as follows:

1. Population initialization: the chromosomes of the GA are initially defined with a logical vector containing five elements: each one of the three hyperparameters and the number of neurons in the two hidden layers. Afterwards, a randomly initialization of the population is performed by assigning a specific value to each particular parameter; in fact, the values assigned to each parameter are within a predefined range of values. Once the initialization of the population is achieved the procedure continues in Step 2.
2. Population assessment: in this step is evaluated the fitness function which is based on the minimization of the reconstruction error between the input and the output features. Specifically, the Equation (1) in Section 2.1 is the GA’s optimization function. Thereby, GA’s aim is to achieve minimum reconstruction error. Therefore, the optimization problem to be solved by the GA involves the search of those specific parameter values that leads a high-performance feature mapping. Then, once the whole population is evaluated under a wide range of values, the condition of best parameter values is analyzed, and the procedure continues in Step 4.
3. Mutation operation: the mutation of the GA produces a new value of the population by means of the roulette wheel selection, the new generated population takes into account the choosing of the best fitness value achieved by the previous evaluated population. Moreover, a mutation operation which is based on the Gaussian distribution is applied during the generation of the new population. Subsequently, the procedure continues in Step 2.
4. Stop criteria: the stop criteria for the GA are two: (i) the obtention of a reconstruction error value lower than a predefined threshold, 5%, and/or, (ii) reaching the maximum number of iterations, 1000 epoch. In the case of the first stop criterion, (i), the procedure is repeated iteratively until those optimal parameter values are found until the GA evolve, then, the procedure continues in Step 3.

Subsequently, the input layer corresponds to the length of the FFT data obtained from the processing, which is used for training the network. The output layer is set to two to generate a two-dimensional feature space. The Adam optimization algorithm that is an extension to stochastic gradient descent, is used to optimize the loss function [48]. The weights are initialized using the Glorot uniform initializer, also called Xavier uniform initializer, which automatically determines the scale of initialization based on the number of input and output neurons [49]. The activation function is set to sigmoid function. Training a DAE is unsupervised, so it does not require label information of the input data. Therefore, the final DAE structure is able to effectively reconstruct the FFT signals used during the training process, and at the same time bottleneck coding layer is able to generate a space of features which can be projected in a two-dimensional space. The encoder output is chosen to be two-dimensional to generate a feature space that can be interpreted by any user. Both functionalities of the DAE framework can be used for anomaly detection. On the one hand, there is the ability to reconstruct signals used during training and the measurement of their reconstruction error. On the other hand, generation of a feature space mapping, on which boundaries can be built and establish anomaly memberships is a possibility. The anomaly memberships depend on the implementation of OCC-based schemas. When working with two-dimensional projections, the anomaly membership can be generated as a boundary in which the known data is enclosed, while the anomalies are outside said membership or boundary. The result of this step is a trained AE model, capable of generating both a feature space, as well as an effective reconstruction of the input signals.

### 3.4. Step 4: Train Deep-Compact Clustering

Although the DAE model has the ability to generate a feature space mapping at the output of the bottleneck coding layer, DAEs may be inefficient when applied directly to OCC problems, since the feature space mapping of the bottleneck can be sparse, i.e., it does not guarantee a compact mapping of the data in the bottleneck, which is an essential issue in OCC problems for a desirable result [36]. In this regard, it is applied as a framework for enhancing the compactness of clusters in the feature space. The deep-compact-clustering introduced in this work follows the idea presented in [40]. Clusters are constructed using the weights learned during the DAE training to initialize a deep neural network with the same architecture of the DAE’s encoder, discarding the decoder part. The objective of DCC is to simultaneously optimize the feature space mapping and the cluster centers through an iterative and unsupervised process. The centers are initialized using fuzzy C-means algorithm, k is a user-defined parameter. In this work, it is established that the k centroids correspond to each one of the known operating conditions (i.e., pairs of torque and speed setpoints considered during the acquisition step) of electromechanical system under study. The DCC model is trained using Adam optimizer with momentum in a standard backpropagation procedure with learning rate 0.001 and batch size is set to 200 is used for all datasets in training stage. The degrees of freedom α of Student’s t-distribution are set in one. For more details refer to Sub-Section 2.2. In this regard, the result of this step is a feature space with compact clusters.

### 3.5. Step 5: Train Oc-Svm

The next step is to carry out the OCC scheme. The clusters compressed in the feature space resulting from the DCC optimization are used to train the OCC scheme. In the pre-sent methodology, the OC-SVM is used as an anomaly classifier. To establish the OC-SVM parameters, a combinatorial search strategy is used [50]. The kernel and regularization parameters are obtained through experimentation. A five-fold cross-validation is used to determine the best results. The RBF kernel is used for all case studies.

Once the parameters are defined and the optimization has converged, the OC-SVM is trained with information of the known scenarios (healthy and faulty sets) but is labeled as a unique class. This means that the model finds a boundary that encloses all the known scenarios, called anomaly membership. A positive value indicates that the given data point is within the hyperplane (it is considered known), on the contrary, a negative value indicates that it is outside the decision boundary (it is considered anomaly). This anomaly membership is the end result of this step.

### 3.6. Step 6: Reconstruction Model

The main idea behind a reconstruction model is to try to recreate the output of a system the same as its input. In this sense, as already discussed in Section 2.1, AEs are models that are trained to successfully learn the optimal mapping of the data to a feature space and also to reconstruct them with small reconstruction error, i.e., $\Omega_{Mse}$. This data can be about the nominal condition of a system. However, the mapping to the feature space is not optimized, thereby, the result is a significantly higher reconstruction error.

In this way, the reconstruction error can be used as a metric to identify outliers or anomalies. Thus, given a test sample $x_{Test}$, this is detected as unknown when the magnitude of its reconstruction error is larger than a certain threshold δ:

(12) $$\Omega_{Mse}=||x_{Test}-y_{Test}{||}^{2}>\delta$$

For the anomaly score, its set a simple threshold $\delta_{95}$ at the 95 percentile of training data distribution. It should be noted that the quality of the data, different operating conditions and the level of fitting to the training data needs careful consideration, as DAEs can also fit to the unknown data. If this is the case, the reconstruction error for anomalies data can be as low as the error for nominal data, which is an undesirable outcome. In this step, an anomaly score based on the reconstruction error measurement is the final result.

### 3.7. Step 7: Decision Rule

At first, the anomaly membership obtained from OC-SVM on the feature space mapping as well as the anomaly score obtained from the reconstruction process could be used as the classification result itself. However, both methods can be misleading for anomaly detection. On the one hand, for anomalous data, the mapping to the feature space is not optimized, despite the cluster compression process, data overlapping can occur on known samples. On the other hand, the measurement of the reconstruction error can fail to detect abnormal samples, as the data considered as anomalies can be fit to the reconstruction values of the nominal data and only those samples with a high error value are perceived outside the threshold and therefore as anomalies. In this regard, in the present methodology, anomaly detection is carried out through the use of the deep feature representation capability of the DAE and then improve the quality of the clusters through the mapping space compaction process in addition to OC-SVM. Then, to strengthen detection, the anomaly score obtained from the DAE’s reconstruction process is used. Therefore, for a given sample X, it is classified as known if the anomaly membership from OC-SVM is positive, which means that the sample is within the boundary. Instead, sample X is classified as abnormal if:

(13) $$\Omega_{Mse}>\delta \;\;\mathrm{or}\;\;AM_{oc-svm}<0$$

where $AM_{oc-svm}$ is the anomaly membership from OC-SVM. Hence, the result of the detection of known samples is given by DCC + OC-SVM, while the identification of anomalies is given by the combination of DCC + OC-SVM and the measurement of $\Omega_{Mse}$ through DAE.

### 3.8. Step 8: Valiadation

In order to evaluate the anomaly detection performance of the DAECC-OC-SVM, the assessment is carried out with the validation and test databases. It has been decided to use true positive (TP) to represent the number of correctly classified known samples, false negative (FN) to represent the number of known samples misclassified as anomaly, true negative (TN) to represent correctly classified anomaly samples and false positive (FP) to represent the number of anomaly samples misclassified as known. The anomaly detection performance is evaluated considering the true positive rate (*TPR*), true negative rate (*TNR*) and the balanced accuracy that refer to recall-based metrics. In addition, precision-based metrics are taken into account, such as, *positive predicted value* (*PPV*) and *negative predictive value* (*NPV*). Are defined below:

(14) $$True\;Positive\;Rate\;\left(TPR\right)=\frac{TP}{TP+FN}$$

(15) $$True\;Negative\;Rate\;\left(TNR\right)=\frac{TN}{TN+FP}$$

(16) Balanced Accuracy=(TPR+TNR)2

(17) $$Positive\;Predictive\;Value\;\left(PPV\right)=\frac{TP}{TP+FP}$$

(18) $$Negative\;Predictive\;Value\;\left(NPV\right)=\frac{TN}{TN+FN}$$

Therefore, the result of the *TP* values is given by the anomaly score obtained through OC-SVM, while the *TN* values are given by the combination of OC-SVM and the resulting samples above the reconstruction model threshold.

## 4. Validation and Analysis

The experimental results are presented in the following order: Section 4.1 presents the experimental electromechanical systems considered to validate the proposed methodology. Next, in Section 4.2, the numerical results are presented. Finally, in Section 4.3, a visual analysis of the results and a discussion for the methodology is presented.

### 4.1. Test Benches Descriptions

To evaluate the effectiveness and adaptability to different electromechanical systems of the proposed methodology, two different experimental test benches have been considered. First, a specific electromechanical system with available acquisition considering faults in different components as well as four different operating conditions. Second, a public test bench focused only on bearing faults, with available acquisition taking into account various bearing fault severities and different load conditions.

#### 4.1.1. Multi-Fault Experimental Test Bench

**Figure 4:**
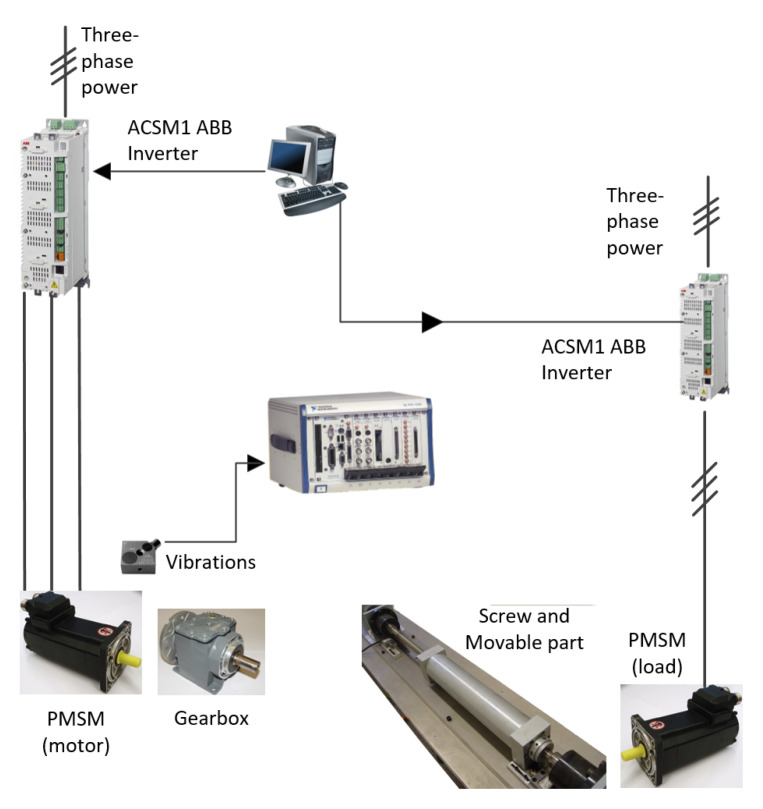
Test bench diagram used for the validation of the anomaly detection-based methodology.

The multi-fault experimental test bench is based on two facing motors with the same characteristics, the motor under monitoring and the motor that works as a load. The two motors are connected by means of a screw and a gearbox. The motor under monitoring runs the input axle of the gearbox. The screw with the movable part is driven by the output axle of the gearbox. The motors are driven by means ABB power converters, ACSM1 model. The motors are two SPMSMs with three pairs of poles, rated torque of 3.6 Nm, 230 Vac, and rated speed of 6000 rpm provided by ABB Group. Vibration signals were acquired with the accelerometer mounted at the motor under test. The measurements were done to a PXIe 1062 acquisition system provided by NI. The sampling frequency was fixed at 20 kS/s during 1 s for each experiment. The experimental test bench diagram is shown in Figure 4. Healthy data (He) and four fault conditions have been considered. The faulty data set are: first, a partially demagnetized motor (Df) was developed during the fabricate with a 50% of nominal flux reduction in one pair of poles. Second, an assembly was carried out with fault in bearings (Bf). The inner and outer races of the non-terminal bearing have been scraped thoroughly in order to cause a generalized rough defect. Third, a static eccentricity (Ef) was induced through a screw attachment in the gearbox output shaft. Fourth, slight degradation is generated on two gear teeth to impose a degree of smoothed on the reduction gearbox (Gf).

The measurements were collected at different operating conditions corresponding to power frequency low, power frequency high (30 and 60 Hz), motor load low and motor load high (40 and 75% of the nominal load). Therefore, there are four resulting operating conditions: power frequency low—motor load low (C1), power frequency low—motor load high (C2), power frequency high—motor load low (C3) and power frequency high—motor load high (C4). For the healthy condition and each of the fault conditions, there are 200 segmented samples based on the acquisitions perform. A total of 200 samples represent a number of valid samples to be obtained during the occurrence of a fault in an operating system [51,52,53].

For this case study, fifteen test scenarios are considered. The distribution of the classes for each scenario is presented in Table 1. In the same way as Section 4.1.1, healthy data and all faulty data are grouped in the following sets: training set, known set and unknown set. Further, and also, it is considered that the healthy condition is initially available and progressively new fault states are detected and incorporated to the diagnosis.

#### 4.1.2. Rolling Bearing Faults Experimental Test Bench

The dataset was acquired from the bearing data center of the Case Western Reserve University [54]. It was collected using accelerometers mounted at the drive end of induction motor which consists of healthy data and faulty data. The faulty data set was generated by single point fault in the ball (FB), the inner race (FI) and the outer race (FO). For each one of the faults, there are three fault sizes corresponding to the various fault severities, 0.007 inches, 0.014 inches and 0.021 inches, respectively. Moreover, the data were collected at different operating conditions corresponding to various motor loads (0, 1, 2, and 3 hp). All data sets were acquired with the sampling frequency of 12 kHz. For each of the conditions considered, such the healthy condition and the fault conditions, there are 200 samples for each one.

Seven scenarios for test are considered to evaluate the capability of the methodology to detect anomaly scenarios and the response of the models to the incorporation of new classes to the initially available information. The distribution of the classes for each scenario is presented in Table 2. The four classes are grouped in three sets: training set, known set and unknown set. Each of the scenarios correspond to a progressing stage of the proposed approach, from an initial knowledge of only the healthy condition (HE) with the four operating conditions, to a scenario where data of three classes is known. These scenarios aim to test the capabilities of the proposed methodology in a real industrial framework where initially the healthy condition is initially available, and progressively new fault states are detected and incorporated. This is done in such a way that one fault state is added to the training stage in each progressing stage.

#### 4.1.3. Datasets Preprocessing and Methodology Adaption

Previous to the proposed method training and posterior validation, an important consideration must be carried out in regard with the model structures to be adapted to the considered experimental data sets:

(1) First, the datasets were divided into three subsets, regarding the proposed methodology. Second, the data were selected randomly and a five-fold cross-validation approach is applied over the data in order to corroborate that the results are statistically significant.
(2) Fast Fourier transform (FFT) is applied to every part of the windowed signals to obtain the corresponding frequency amplitude. The frequency amplitude of sample is scaled following the recommendations of [46].
(3) For the multi-fault experimental test bench, the data of the plane perpendicular to the motor rotation are used. While for the bearing fault dataset, it takes the single-sided frequency amplitude calculated in the last step as the final input for the model. Therefore, the input dimension for the experimental test bench for the length of the input is 2048, and for the rolling bearing dataset, it is 1024.
(4) Three autoencoders were used to build the deep-autoencoder. The hidden layers were established through search optimization using a GA. For the experimental test bench, the architecture of the deep-autoencoder is d -850-120-2, for the encoder part, where d is the dimension of input data. The decoder is a mirror of the encoder with dimensions 2-120-850- d. While the encoder architecture of DAE for the rolling bearing dataset is d-500-100-2, also d is the dimension of input data.

### 4.2. Experimental Results

In order to verify the effectiveness and performance of the proposed DAECC-OC-SVM method, the results obtained are compared with three representative unsupervised anomaly methods. First a reference method and additionally two variant simples of the DAECC-OC-SVM:

(1) Reference method: reconstruction-model with deep-autoencoder [33,55];
(2) Variant simple Method 1: deep-autoencoder + OC-SVM;
(3) Variant simple Method 2: deep compact clustering + OC-SVM.

(1) is a reconstruction-based method as described in Section 3.6. In this, only a threshold is used as a metric to identify anomalies. This method has been successfully implemented in different applications. (2) is a method that integrates a deep-autoencoder model without the improved feature space compaction process and the anomaly detection method based on a one-class support-vector machine. (3) is a simplified version of the DAECC-OC-SVM method (the reconstruction model is not taken into account).

#### 4.2.1. Results on Multi-Fault Experimental Test Bench

The results of the application of the proposed methodology, the DAECC-OC-SVM, as well as the comparison with the reference methods are detailed in Table 3 and Table 4. Results are displayed first in terms of recall-based metrics such as *TPR* and *TNR* for each of the test scenarios. In this regard, it can be observed that in the case of *TPR*, that is, the known cases, the best performance obtained is through the DAE-MSE method, as it is higher in thirteen of the fifteen scenarios (S1, S3–S14). However, the anomaly detection capabilities of this method are quite limited, being the worst method for this task, being superior in only two scenarios (S9 and S15) and having poor performance in most scenarios. Otherwise, the combination of DAE + OC-SVM presents unsatisfactory results, standing out only in a scenario of *TPR* (S2). The most of *TPR* are large whereas its *TNR* in most cases is less than 0.50, which indicates that most of the samples of unknown classes cannot be detected. Moreover, with the compaction of the feature space through DCC, the detection of anomalies is considerably improved. As shown in Table 3, the *TNR* values increase relative to the uncompressed method.

In this regard, the proposed methodology adopts the capabilities of DCC to improve the compactness of the clusters in the feature space and thus increase the OC-SVM detection performance. Furthermore, by combining the DAE reconstruction function, it is possible to improve the accuracy of anomalous cases. Therefore, the proposed method, DAECC-OC-SVM outperforms other listed methods in the detection of anomaly samples in each of the scenarios considered.

In Table 4 shows the corresponding balanced accuracy. It should be noted that the DAECC-OC-SVM based method is superior to the other methods in each of the scenarios. It only coincides in accuracy in four scenarios (S6, S8, S13, S14) with the simplified version, that is, DCC + OC-SVM. This means that the reconstruction model (DAE MSE) does not provide any improvement when it comes to detecting abnormal samples in these four scenarios. In percentage terms, the DAECC-OC-SVM method obtains an average of the fifteen scenarios of 87.70%, and is higher by 21.70%, 28.05% and 12.10%, compared to Methods (1), (2) and (3), correspondingly.

Furthermore, Table 5 shows the results for the detection of anomalies based on precision, that is, *PPV* and *NPV*. It can be noted that the DAECC-OC-SVM proposal is superior to the rest of the anomaly detection methods. In terms of *PPV*, DAECC-OC-SVM scores the best in all fifteen test scenarios. Whereas in terms of *NPV*, it is superior in twelve out of fifteen test scenarios. In percentage terms, the DAECC-OC-SVM method obtains an average of the fifteen scenarios of 94.60% for *PPV*, and 73.80% for *NPV*. DAECC-OC-SVM is higher by 12.30%, 16.70% and 7.70% for *PPV* and is higher by 30.80%, 24.67% and 14.32%, for *NPV*, compared to method (1), (2) and (3), correspondingly.

#### 4.2.2. Results on Bearing Fault Experimental Test Bench

The results of DAECC-OC-SVM and the other methods on rolling bearing dataset are detailed in Table 6. According to the results obtained in terms of *TPR* and *TNR* shown in Table 5, it can be seen that the data corresponding to the detection of anomalies (*TNR*) of DAECC-OC-SVM outperforms the other listed methods. While the results obtained from TPR, the proposed method is superior in four out of seven analysis scenarios (SS1, SS5, SS6 and SS7). The DAE without the DCC compression enhancement achieves the best performance of the remaining three *TPR* scenarios. However, this method achieves the worst results for detecting unknown cases.

According to the results calculated in Table 7, i can be seen that the balanced accuracy of DAECC-OC-SVM reaches 97.6% (in percentage terms), and this result outperforms other listed methods. Specifically, the balanced accuracy of the DAE-MSE method is 88.2%, which is 9.4% lower than DAECC-OC-SVM. The DAE + OC-SVM method obtains a balanced accuracy of 74.5%, while DCC + OC-SVM obtains 93.8% as a result.

The proposed method outperforms by 23.1% and 3.8%, respectively, than the corresponding methods. It should be noted that, for each of the seven analysis scenarios, DAECC-OC-SVM is superior to other methods. Only in the SS1 scenario, DCC + OC-SVM obtains the same accuracy as the proposed method.

Moreover, in Table 8 shows the results for the detection of anomalies based on precision. It can be noted that the DAECC-OC-SVM proposal is superior to the rest of the anomaly detection methods. In terms of *PPV*, DAECC-OC-SVM scores the best in all seven test scenarios. In the same way, in terms of *NPV*, it is superior in all seven test scenarios.

### 4.3. DAECC-OC-SVM Performance Discussion

In order to give an understanding of the effectiveness of anomaly detection method proposed, some additional tests are presented to show behavior and performance. For this purpose, first, an analysis of the core of the methodology is performed, that is, the capabilities of characterization and representation of DAE. A DAE is optimized by minimizing the reconstruction error between input and output.

**Figure 5:**
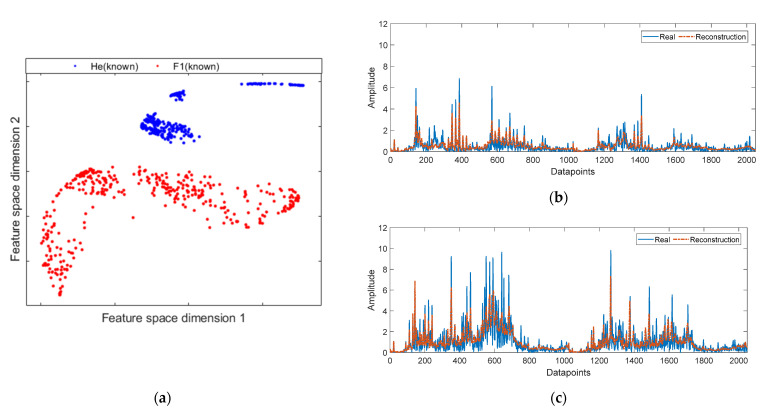
Characterization of the healthy condition and a fault condition by mean DAE: (**a**) feature space mapping of the bottleneck obtained through a traditional DAE; (**b**) real and reconstructed healthy condition signal; (**c**) real and reconstructed signal of a fault condition.

Furthermore, in this process, it maps similar features close to each other in the bottleneck feature space, at the output of the encoder process. On the contrary, samples that are different are mapped to distant spaces. As can be seen in Figure 5a, where the feature mapping, obtained from a simple DAE model, presents the corresponding clusters for the healthy state (He) and a fault condition (F1). It should be noted that each health state presents different sets of clusters, this is due to the different operating conditions of the experimental test bench. Besides, in Figure 5b,c, a sample of each of the health states, He and F1, correspondingly, and its resulting reconstruction obtained by DAE after training, are shown. Qualitatively, it can be seen that the signals reconstructed through DAE have a high similarity with their corresponding real signal. Although it is not an exact reproduction, the reconstruction follows the form of the most representative harmonics for each of the conditions. Those differences that occur, that is, changes in aptitude, such as increases or decreases, or changes in the position of the harmonics are due to the intrinsic properties of the system, such as nonlinearities, as well as the presence of noise, oscillations, or external interferences. In terms of reconstruction error, the He sample has a $\Omega_{Mse}$ of 0.140, while the F1 sample, corresponding to a Bf. has an $\Omega_{Mse}$ of 0.710. These $\Omega_{Mse}\;$ values are consistent for each corresponding health states.

**Figure 6:**
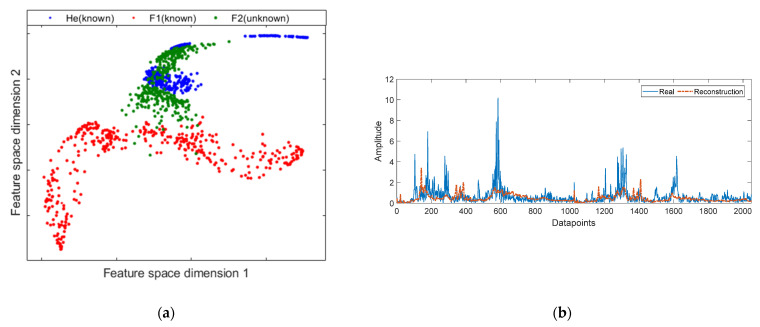
Characterization of known states (He and F1) and an unknown fault state (F2): (**a**) feature space mapping of the bottleneck obtained through a traditional DAE; (**b**) real signal of unknown fault status and its respective reconstruction by mean DAE.

However, when trying to characterize a sample of a novel fault condition that would be interpreted as an anomaly, the traditional DAE model can have certain drawbacks. Due to the DAE learning process being unsupervised, the representation of the data in the feature space is not optimized, therefore, there is no compact representation. The hypothesis is that the compact feature space increases the separability between normal and abnormal states. Therefore, the abnormal samples could be very close to or overlap with the normal samples. This effect is shown in Figure 6a, where a significant overlap between normal and abnormal samples mapped by the DAE’s bottleneck is observed. The abnormal samples are represented by F2 which corresponds to a fault by Gf.

Furthermore, it is important to note that the mapping feature learned by a DAE is specific to the training data distribution, i.e., a DAE will typically not succeed at reconstructing data which is significantly different from data it has seen during training. However, the quality of the data can influence the measurement of the reconstruction error. Since the DAE mapping can also fit the unknown data, producing a reconstruction error similar to the training data. In this sense, Figure 6b shows the characterization of a fault sample (F2), not seen in DAE training. Similarly, qualitatively it can be noted that a sample not seen in the fit of the DAE is not reconstructed effectively.

For example, in Figure 6b, the reconstructed signal is not similar to the harmonics corresponding to the 600 datapoints. In terms of reconstruction error, the sample from the F2 data set has an MSE of 0.69. This MSE value is clearly higher than the He state, but it is slightly lower than the F1 fault. Therefore, although in some cases, setting a novelty threshold would be enough to detect uncharacterized fault samples, in this case study, establishing a threshold would not be successful with some fault states, because the MSE of the anomalous samples is lower than that of some samples of training.

**Figure 7:**
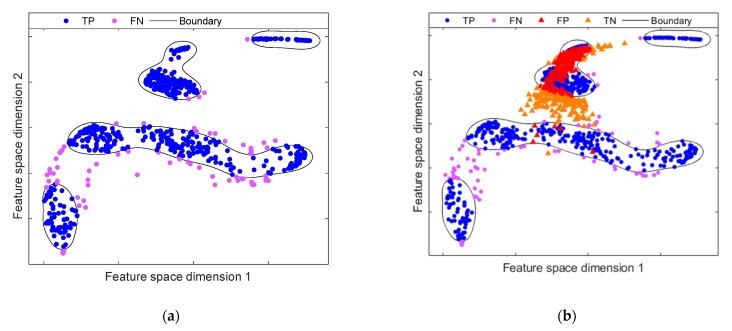
Anomaly detection model on feature space mapping resulting from DAE by means OC-SVM: (**a**) initial anomaly model representation. The anomaly model is trained employing data from He condition and F1 condition; (**b**) evaluation of the fault scenario F2, showing a high overlap between known and unknown classes.

**Figure 8:**
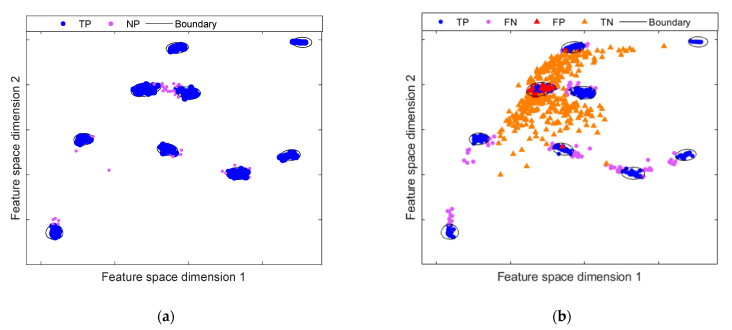
Anomaly detection model on compact feature space mapping resulting from DCC model by means OC-SVM: (**a**) initial anomaly model representation with compact features; (**b**) evaluation of the fault scenario F2; the compact representation of the normal class leads to a higher separation between the anomaly class, which results in a greater number of correctly classified samples, compared to traditional DAE.

To address these shortcomings for anomaly detection, the DCC is introduced to improve the feature rendering resulting from the DAE’s bottleneck. The objective of DCC is to achieve a compact representation of the feature space mapping. As mentioned, a traditional DAE is not optimized to generate a compact representation of the data, producing overlap between normal and abnormal samples. This overlap hinders the performance of the classifier. As shown in Figure 7a, the boundary created through the OC-SVM scheme is too lengthy for normal data, due to the feature space is not dense. In this regard, when projecting the data of the anomalous samples in the mapped space, these will be placed within the boundary, producing high values of false negatives. This effect is shown in Figure 7b. A significant overlap can be noted between normal and abnormal samples mapped within the boundary generated by OC-SVM. In contrast, when the DCC introduces compactness in the representation and denser clusters are produced, the OC-SVM scheme generates close boundaries, as shown in Figure 8a. Consequently, there is a significant reduction in the overlapping areas between normal samples and abnormal samples, as seen in Figure 8b. Note that the representation of normal samples has been grouped into several very compact groups compared to the mapping provided by the traditional DAE. This is owing to the fact that centroids provide the distribution of the data, corresponding to the different operating conditions of the system.

## 5. Conclusions

In this paper, DAECC-OC-SVM, a novel method for anomaly detection in industrial electromechanical systems, is proposed. DAECC-OC-SVM is based, first, on the high pat-tern management of deep-learning to characterize and extract features from complex industrial systems that are usually incorporated in smart manufacturing environments. Second, the application of a deep-clustering algorithm to improve feature space mapping for learning compact representations. Third, this compact representation is used as input to a one-class classification scheme, demonstrating that the feature space mapping com-paction improves the implementation of these schemes when outliers or anomalies are addressed. Finally, the combination with the reconstruction capacity of the DAE, allows to improve the detection of outliers.

In order to demonstrate the effectiveness of the methodological proposal, two experimental systems are analyzed to carry out the validation: a multi-fault experimental test bench and a bearing fault experimental test bench. The multi-fault experimental test bench is driven by a permanent-magnet synchronous motor, while the bearing fault experimental test bench by an induction motor. Therefore, the proposed method is not limited to the use of a single engine technology. Based on all experimental studies, the effectiveness of the developed methodology under different operating conditions (load and speed) and different healthy conditions is demonstrated.

The results are analyzed first quantitatively and then qualitatively. The proposed method is compared with three different anomaly detection schemes. The first, a reconstruction model based on a deep-autoencoder, for this case an anomaly threshold is established for samples with a high reconstruction error. The second, a model based on feature space mapping through a DAE without the compaction process. The third, a simplified version of the proposed method without taking into account the combination with the re-construction model. The achieved results demonstrate the superiority of the proposed over other anomaly detection schemes since through this proposal an average of 87.7% for the multi-fault experimental test bench and an average 97.6% for the bearing fault experimental test bench is reached for the classification ratio. For the fifteen test scenarios of the multi-fault experimental test bench, the proposed method is superior in fourteen, obtaining the best average compared to the other methods.

While in the seven scenarios of the bearing fault experimental test bench, the proposed method is more effective than the existing methods.

One of the main advantages of the DAECC-OC-SVM proposal is that it is adaptable to any electromechanical system in which an anomaly detection is carried out. The high effectiveness of the proposed model demonstrates its viability in the application of complex industrial environments. However, and as in most data-based approaches, the effectiveness of the model depends largely on the quality of the data, the ability of the model to generate compact clusters and its distribution in the feature space.

The detection of anomalies is a difficult task to face and is far from being solved. In the field of monitoring the condition of industrial systems, the detection of anomalies must face different problems, such as the physical configuration of the system, the different operating conditions and the presence of different faults, and therefore, must be a tool that allows solving inconveniences presented in the production processes. In this regard, the present work proposed a methodological process for the detection of anomalies in electromechanical systems. The contributions presented in this work can be applied to re-al world settings and therefore generate additional research.

## Figures and Tables

**Table 1:** Experimental set for each training and testing scenario.

Label	Training Set	Testing Set	
		Known Set	Unknown Set
S1	He	He	Bf, Df, Ef, Gf
S2	He, Bf	He, Bf	Df, Ef, Gf
S3	He, Df	He, Df	Bf, Ef, Gf
S4	He, Ef	He, Ef	Bf, Df, Gf
S5	He, Gf	He, Gf	Bf, Df, Ef
S6	He, Bf, Df	He, Bf, Df	Ef, Gf
S7	He, Bf, Ef	He, Bf, Ef	Df, Gf
S8	He, Bf, Gf	He, Bf, Gf	Df, Ef
S9	He, Df, Ef	He, Df, Ef	Bf, Gf
S10	He, Df, Gf	He, Df, Gf	Bf, Ef
S11	He, Ef, Gf	He, Ef, Gf	Bf, Df
S12	He, Bf, Df, Ef	He, Bf, Df, Ef	Gf
S13	He, Bf, Df, Gf	He, Bf, Df, Gf	Ef
S14	He, Bf, Ef, Gf	He, Bf, Ef, Gf	Df
S15	He, Df, Ef, Gf	He, Df, Ef, Gf	Bf

**Table 2:** Experimental set for each training and testing scenario for the rolling bearing dataset.

Label	Training Set	Testing Set	
		Known Set	Unknown Set
SS1	HE	HE	FB, FI, FO
SS2	HE, FB	HE, FB	FI, FO
SS3	HE, FI	HE, FI	FB, FO
SS4	HE, FO	HE, FO	FB, FI
SS5	HE, FB, FI	HE, FB, FI	FO
SS6	HE, FB, FO	HE, FB, FO	FI
SS7	HE, FI, FO	HE, FI, FO	FB

**Table 3:** Comparison of performance of anomaly detection in terms of *TPR* and *TNR* on multi-fault test bench.

Label	DAE-MSE		DAE + OC-SVM		DCC + OC-SVM		DAECC-OC-SVM	
	TPR	TNR	TPR	TNR	TPR	TNR	TPR	TNR
S1	**0.982**	0.607	0.891	0.409	0.936	0.662	0.936	**0.894**
S2	0.908	0.003	**0.931**	0.573	0.862	0.797	0.862	**0.801**
S3	**0.988**	0.672	0.888	0.145	0.908	0.542	0.908	**0.877**
S4	**0.933**	0.770	0.911	0.138	0.873	0.677	0.873	**0.922**
S5	**0.983**	0.370	0.892	0.402	0.869	0.558	0.869	**0.874**
S6	**0.922**	0.020	0.917	0.459	0.852	**0.797**	0.852	**0.797**
S7	**0.920**	0.013	0.909	0.278	0.870	**0.811**	0.870	**0.811**
S8	**0.937**	0.000	0.910	0.496	0.916	**0.830**	0.916	**0.830**
S9	**0.967**	0.993	0.929	0.035	0.911	0.291	0.911	**0.997**
S10	**0.967**	0.500	0.930	0.17	0.934	0.435	0.934	**0.851**
S11	**0.949**	0.611	0.917	0.02	0.873	0.721	0.873	**0.887**
S12	**0.936**	0.030	0.924	0.322	0.879	0.800	0.879	**0.802**
S13	**0.938**	0.000	0.932	0.228	0.835	**0.822**	0.835	**0.822**
S14	**0.938**	0.000	0.923	0.262	0.860	**0.782**	0.860	**0.782**
S15	0.954	**1.000**	0.936	0.000	**0.973**	0.001	**0.973**	**1.000**

**Table 4:** Average balanced accuracy over 15 experimental scenarios with different methods.

Label	Balanced Accuracy			
	DAE MSE	DAE + OC-SVM	DCC + OC-SVM	DAECC-OC-SVM
S1	0.795	0.648	0.799	**0.915**
S2	0.456	0.752	0.830	**0.831**
S3	0.830	0.516	0.725	**0.892**
S4	0.851	0.524	0.775	**0.897**
S5	0.677	0.647	0.713	**0.871**
S6	0.476	0.688	**0.835**	**0.835**
S7	0.469	0.593	0.805	**0.840**
S8	0.461	0.703	**0.873**	**0.873**
S9	**0.979**	0.482	0.601	0.954
S10	0.736	0.550	0.653	**0.892**
S11	0.783	0.468	0.765	**0.880**
S12	0.483	0.623	0.839	**0.840**
S13	0.469	0.580	**0.828**	**0.828**
SS4	0.469	0.592	**0.821**	**0.821**
S15	0.977	0.468	0.487	**0.986**
Average	0.660	0.597	0.756	**0.877**

**Table 5:** Comparison of performance of anomaly detection in terms of *PPV* and *NPV* on the multi-fault test bench.

Label	DAE MSE		DAE + OC-SVM		DCC + OC-SVM		DAECC-OC-SVM	
	PPV	NPV	PPV	NPV	PPV	NPV	PPV	NPV
S1	0.777	**0.947**	0.595	0.760	0.740	0.909	**0.907**	0.932
S2	0.646	0.000	0.857	0.821	**0.908**	**0.720**	**0.908**	**0.720**
S3	0.888	0.860	0.677	0.415	0.763	0.704	**0.934**	**0.821**
S4	0.909	0.820	0.682	0.461	0.834	0.721	**0.957**	0.784
S5	0.783	0.578	0.789	0.709	0.870	0.609	**0.981**	**0.745**
S6	0.740	0.101	0.869	0.704	**0.992**	**0.644**	**0.922**	**0.644**
S7	0.739	0.085	0.800	0.563	0.920	0.670	**0.922**	**0.672**
S8	0.734	0.000	0.836	0.636	**0.938**	**0.777**	**0.938**	**0.777**
S9	0.997	**0.968**	0.746	0.195	0.791	0.426	**0.998**	0.796
S10	0.872	0.457	0.792	0.541	0.811	0.334	**0.942**	**0.745**
S11	0.894	0.755	0.740	0.129	0.827	0.605	**0.940**	**0.763**
S12	0.790	0.028	0.845	0.516	0.954	0.633	**0.955**	**0.634**
S13	0.789	0.000	0.828	0.460	**0.974**	**0.557**	**0.974**	**0.557**
S14	0.789	0.000	0.833	0.462	**0.916**	**0.598**	**0.916**	**0.598**
S15	**1.000**	0.840	0.789	0.000	0.795	0.018	**1.000**	**0.885**

**Table 6:** Comparison of performance of anomaly detection in terms of *TPR* and *TNR* on CWRU dataset.

Label	DAEH MSE		DAE + OC-SVM		DCC + OC-SVM		DAECC-OC-SVM	
	TPR	TNR	TPR	TNR	TPR	TNR	TPR	TNR
SS1	0.879	**1.000**	0.986	0.871	**0.997**	0.999	**0.997**	**1.000**
SS2	0.950	0.862	**0.984**	0.376	0.963	0.884	0.963	**0.980**
SS3	0.950	0.558	**0.998**	0.732	0.992	0.874	0.992	**0.937**
SS4	0.950	0.854	**0.985**	0.552	0.964	0.960	0.964	**0.983**
SS5	0.950	0.785	0.948	0.015	**0.987**	0.923	**0.987**	**0.929**
SS6	0.950	**1.000**	0.891	0.594	**0.982**	0.690	**0.982**	**1.000**
SS7	0.950	0.718	0.930	0.583	**0.960**	0.970	**0.960**	**0.995**

**Table 7:** Average balanced accuracy over seven scenarios on the CWRU dataset.

Label	Balanced Accuracy			
	DAE MSE	DAE + OC-SVM	DCC + OC-SVM	DAECC-OC-SVM
SS1	0.939	0.928	**0.998**	**0.998**
SS2	0.906	0.680	0.923	**0.972**
SS3	0.754	0.865	0.933	**0.965**
SS4	0.902	0.768	0.962	**0.973**
SS5	0.868	0.481	0.955	**0.958**
SS6	0.975	0.743	0.836	**0.991**
SS7	0.834	0.756	0.965	**0.978**
Average	0.882	0.745	0.938	**0.976**

**Table 8:** Comparison of performance of anomaly detection in terms of *TPR* and *TNR* on CWRU dataset.

Label	DAE MSE		DAE + OC-SVM		DCC + OC-SVM		DAECC-OC-SVM	
	PPV	NPV	PPV	NPV	PPV	NPV	PPV	NPV
SS1	1.000	0.892	0.912	0.963	0.999	**0.997**	**1.000**	**0.997**
SS2	0.936	0.893	0.782	0.885	0.944	0.922	**0.990**	**0.930**
SS3	0.834	0.767	0.896	0.991	0.940	0.984	**0.970**	**0.985**
SS4	0.933	0.892	0.860	0.932	0.980	0.931	**0.991**	**0.933**
SS5	0.930	0.839	0.742	0.088	0.974	**0.961**	**0.976**	**0.961**
SS6	1.000	0.869	0.868	0.647	0.905	0.930	**1.000**	**0.950**
SS7	0.910	0.827	0.870	0.735	0.989	0.892	**0.998**	**0.894**

## Data Availability

The data referring to Section 4.1.1. They are own data, so the property is protected and they are not public. The data referring to Section 4.1.2 correspond to the data center of the Case Western Reserve University. It is a public database that can be found at: https://csegroups.case.edu/bearingdatacenter/pages/welcome-case-western-reserve-university-bearing-data-center-website (accessed on 1 August 2021).
